# The Change of Skeletal Muscle Caused by Inflammation in Obesity as the Key Path to Fibrosis: Thoughts on Mechanisms and Intervention Strategies

**DOI:** 10.3390/biom15010020

**Published:** 2024-12-27

**Authors:** Yixuan Li, Wenwen Guo, Han Li, Yuhao Wang, Xinwei Liu, Wen Kong

**Affiliations:** 1 Department of Endocrinology, Union Hospital, Tongji Medical College, Huazhong University of Science and Technology, Wuhan 430022, China; 2 Diabetes and Metabolic Disease Clinical Research Center of Hubei Province, Wuhan 430022, China; 3 Hubei Key Laboratory of Metabolic Abnormalities and Vascular Aging, Huazhong University of Science and Technology, Wuhan 430022, China; 4 Hubei Branch of National Center for Clinical Medical Research of Metabolic Diseases, Wuhan 430022, China

**Keywords:** obesity, skeletal muscle, inflammation, fibrosis, insulin resistance

## Abstract

Obesity leads to a chronic inflammatory state throughout the body, with increased infiltration of immune cells and inflammatory factors in skeletal muscle tissue, and, at the same time, the level of intracellular mitochondrial oxidative stress rises. Meanwhile, obesity is closely related to the development of skeletal muscle fibrosis and can affect the metabolic function of skeletal muscle, triggering metabolic disorders such as insulin resistance (IR) and type 2 diabetes (T2D). However, whether there is a mutual regulatory effect between the two pathological states of inflammation and fibrosis in obese skeletal muscle and the specific molecular mechanisms have not been fully clarified. This review focuses on the pathological changes of skeletal muscle inflammation and fibrosis induced by obesity, covering the metabolic changes it causes, such as lipid deposition, mitochondrial dysfunction, and dysregulation of inflammatory factors, aiming to reveal the intricate connections between the two. In terms of intervention strategies, aerobic exercise, dietary modification, and pharmacotherapy can improve skeletal muscle inflammation and fibrosis. This article provides insight into the important roles of inflammation and fibrosis in the treatment of obesity and the management of skeletal muscle diseases, aiming to provide new ideas for the diagnosis and treatment of metabolic diseases such as obesity and IR.

## 1. Introduction

Obesity has become a global public health issue affecting human health. Due to lifestyle changes and dietary adjustments, such as a sedentary lifestyle and consuming large amounts of processed meats, refined grains, and sugary drinks, the prevalence of obesity has significantly increased. The chronic systemic inflammation caused by obesity may lead to insulin resistance (IR) and β-cell dysfunction, ultimately resulting in type 2 diabetes (T2D) [1]. Thus, obesity is a major driver of the T2D epidemic. This chronic inflammation also significantly raises the risk of long-term diabetes complications like cardiovascular diseases, kidney diseases, non-alcoholic fatty liver disease (NAFLD), stroke, and peripheral vascular diseases, and may underlie the association between T2D and other diseases such as polycystic ovary syndrome, gout, Alzheimer’s disease, and osteoarthritis [2]. In the past three decades, the number of people with diabetes worldwide has quadrupled, with 90% being T2D, and diabetes has become the ninth leading cause of death [3].

**Figure 1:**
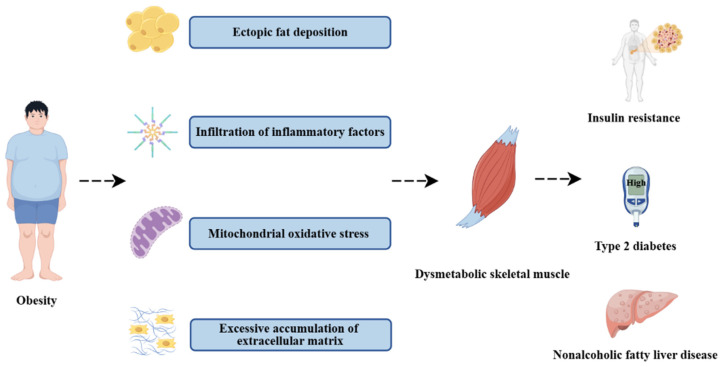
Effects of obesity on skeletal muscle metabolism (by Figdraw). In the obese state, skeletal muscle undergoes tissue remodeling through ectopic lipid deposition, inflammatory responses, mitochondrial oxidative stress, and collagen fiber accumulation, resulting in metabolic disorders that in turn disrupt systemic glucose homeostasis, and are closely associated with the development of insulin resistance, type 2 diabetes mellitus, and nonalcoholic fatty liver disease.

Skeletal muscle, as the principal target organ for glucose uptake upon insulin stimulation, serves as the primary driving force for controlling systemic blood glucose and enhances its sensitivity to insulin stimulation via muscle contraction [4]. Additionally, as an endocrine organ, skeletal muscle generates and secretes a substantial number of bioactive molecules known as “myokines”, which function as autocrine, paracrine, or endocrine intercellular signal transduction molecules and play a crucial role in regulating skeletal muscle mass, transforming muscle fibers, and modulating glucose and lipid metabolism [5]. Abnormalities in skeletal muscle metabolism can significantly impact systemic glucose homeostasis and insulin sensitivity. Recent research has revealed that obesity can lead to the infiltration of immune cells in skeletal muscle, as well as the pro-inflammatory activation of adipose tissue between and around muscle cells, thereby inducing inflammation in muscle cells [6]. Moreover, ectopic lipid deposition [7], an increase in intracellular mitochondrial oxidative stress levels [8], and metabolic disorders in muscle cells can occur, resulting in IR. Concurrently, studies have demonstrated that in the skeletal muscle of obese mice induced by a high-fat diet (HFD), an increase in the level of inflammatory factors is accompanied by an increase in the degree of fibrosis [9]. Consequently, as shown in Figure 1, obesity can trigger poor metabolic function in skeletal muscle tissue and is closely associated with the occurrence and development of IR and T2D.

However, at present, the relationship between skeletal muscle inflammation and fibrosis induced by obesity remains incompletely understood, and relevant research still requires further in-depth exploration. Consequently, this paper deeply explores the relationship between skeletal muscle inflammation and fibrosis due to obesity, focusing on the specific mechanisms of their occurrence and development, providing a theoretical basis for uncovering the pathogenesis of obesity-related diseases and seeking preventive and treatment measures.

## 2. The Development of Skeletal Muscle Inflammation Caused by Obesity

### 2.1. Lipid Deposition

**Figure 2:**
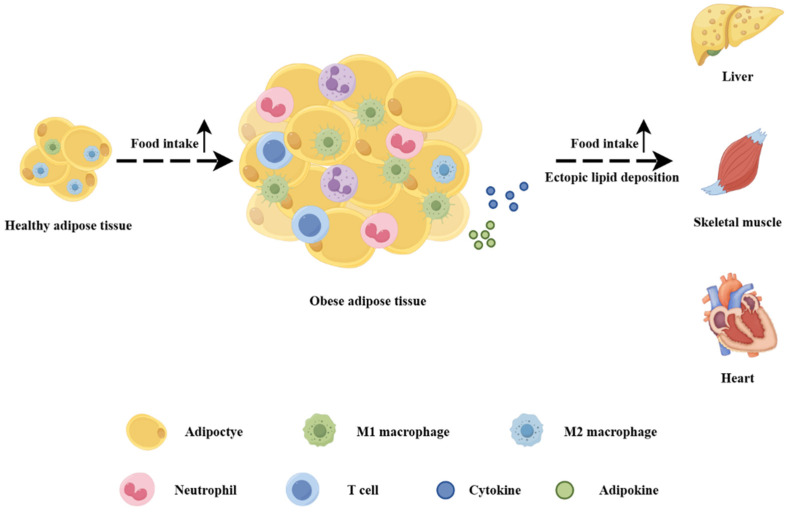
Effects of obesity on adipose tissue (by Figdraw). Adipose tissue in the obese state compensates by increasing adipocyte volume and increasing adipocyte number, while an inflammatory response occurs, as evidenced by M1 macrophage predominance and increased secretion of proinflammatory cytokines and adipokines. With further increases in fat content, ectopic lipid deposition occurs in the liver, skeletal muscle, and heart, leading to metabolic disorders.

An increasing amount of evidence indicates that obesity can trigger inflammatory responses in skeletal muscle and exert a negative regulatory effect on muscle cell metabolism [6]. In the state of obesity, the elevation of fat content polarizes macrophages in adipose tissue towards the M1 phenotype, augmenting the expression of pro-inflammatory cytokines and adipokines, thereby inducing systemic low-grade inflammation and resulting in the emergence of metabolic syndrome (MS), IR, and T2D [1]. As shown in Figure 2, although obese adipose tissue compensates by altering the volume (hypertrophy) and number (hyperplasia) of adipocytes, its fat-storing capacity is limited. Excessive energy intake by the body leads to the overproduction of free fatty acids [10], causing abnormal lipid deposition in tissues, such as the liver, skeletal muscle, and heart, and instigating chronic tissue inflammation [6,7,11]. When lipids and their derivatives are deposited within the muscle, the capacity for cellular mitochondrial β-oxidation is impaired with an increase in reactive oxygen species production to create a lipotoxic environment and concurrently the secretion of some pro-inflammatory myokines is enhanced [8]. At this point, skeletal muscle, which is responsible for processing the majority of glucose, experiences metabolic dysregulation, thereby influencing whole-body homeostasis.

### 2.2. Mitochondrial Dysfunction

Researchers have witnessed an elevated phosphorylation level of AMPK in the soleus [12] and gastrocnemius [13] muscles of HFD-induced obese mice. It is known that AMPK activation stimulates mitochondrial gene transcription mediated by PPAR delta and PGC-1α. Under obese conditions, the augmented inflammatory signals and lipotoxicity can undermine AMPK signaling within skeletal muscle, thereby reducing mitochondrial biogenesis [14]. This, in turn, results in a decline in the mitochondrial oxidative capacity of skeletal muscle cells and triggers the accumulation of fatty acids [15]. In obese rats, the mitochondrial function of the quadriceps femoris is impaired [16], accompanied by excessive accumulation of fatty acids, as well as enhanced inflammatory responses and oxidative stress [10]. In the gastrocnemius muscle of obese mice, not only are the expression levels of inflammatory factors (such as tumor necrosis factor α (TNF-α), Toll-like receptor 2 (TLR2), Toll-like receptor 4 (TLR4), and monocyte chemoattractant protein 1 (MCP1)) elevated, but also the levels of fatty acids, triglycerides, and cholesterol in the muscle are significantly increased [13]. Moreover, the increased fatty acids, lipopolysaccharides, and TNF-α can reduce the activities of protein kinase B (AKT) and AMPK [15], and, consequently, obesity ultimately leads to a reduction in the glucose uptake and lipid oxidation capacity of skeletal muscle, thereby exacerbating IR.

Simultaneously, in a study of obese individuals, it has been demonstrated that as the body mass index rises, the mitochondrial respiration and oxidative capacity of skeletal muscle decrease, which is associated with elevated levels of inflammatory molecules such as C-reactive protein (CRP), TNF-α, interferon-gamma (IFN-γ), and interleukin-8 (IL-8) in the circulation [17]. Additionally, research has revealed that the functions of the immunoproteasome and total proteasome in obese muscles are significantly diminished and that there is a negative correlation between the proteasome function and the level of oxidatively damaged proteins in the muscle, indicating that immunoproteasome (iProt) dysregulation might be a crucial factor contributing to muscle oxidative stress [18]. This dysregulation can damage proteins within the muscle, endanger muscle integrity, and lead to obesity-related IR. Collectively, both animal and human studies suggest that the systemic inflammation caused by obesity is associated with impaired mitochondrial function in skeletal muscle.

In HFD rats, a shift of skeletal muscle energy substrate distribution towards fatty acid oxidation has been observed and muscles with higher fatty acid oxidation rates are rich in intermediate lipids like diacylglycerol (DAG) and ceramide [19]. Thus, obesity-induced skeletal muscle mitochondrial dysfunction leads to more significant intermediate lipid accumulation, exacerbating metabolic dysfunctions such as IR.

### 2.3. Insulin Resistance

Glucose transporter type 4 (GLUT4), the principal glucose transporter in skeletal muscle, and AKT, the primary mediator of insulin-induced GLUT4 translocation to the plasma membrane, play crucial roles in glucose metabolism, and the activation of the insulin receptor substrate 1 (IRS-1)/phosphoinositide 3-kinase (PI3K)/AKT promotes glucose uptake within skeletal muscle [20,21]. A reduction in AKT phosphorylation can trigger IR, thereby decreasing glucose uptake [22]. Given that skeletal muscle is the main site of postprandial glucose uptake, accounting for about 70–80% of whole-body glucose uptake, decreased glucose uptake can lead to hyperglycemia [23,24]. Moreover, the compensatory elevation in insulin secretion, which is stimulated by skeletal muscle IR, can further induce β-cell hypertrophy and hyperinsulinemia [25].

The nuclear factor kappa-B (NF-κB) signaling has been demonstrated to contribute to the development of IR and systemic chronic inflammation by facilitating the transcription of pro-inflammatory cytokines and chemokines [26]. Research has revealed that in individuals with IR (including those with obesity and T2D), the gene expression of interleukin-6 (IL-6) in skeletal muscle is enhanced, accompanied by an augmented activation of the NF-κB pathway [27,28]. Concurrently, in the soleus muscle of HFD-induced obese mice, NF-κB nuclear translocation and an increased level of kappa B (IκB) phosphorylation have been observed [12]. In the skeletal muscle of obese mice, it has been found that inflammation is increased through the NF-κB signaling pathway with the manifestation of inflammatory factors’ (elevated levels of interleukin-1β (IL-1β) and NF-κB proteins) infiltration, while the activation of the IRS-1/PI3K/AKT signaling pathway is weakened and the expression and translocation of GLUT4 are inhibited [29]. Therefore, the muscle’s glucose uptake ability is reduced, the skeletal muscle’s metabolic function is disrupted, and, finally, IR occurs.

### 2.4. Other Metabolic Alterations

Besides being associated with lipid deposition, mitochondrial oxidative stress, and IR, the inflammatory response of skeletal muscle induced by obesity also exerts an impact on the thermogenic capacity of brown adipose tissue. Brown adipose tissue engages in energy balance through thermogenesis mediated by uncoupling protein-1 (UCP-1), while inflammatory cytokines and lipotoxicity can decrease the UCP-1 activity and thermogenic potential of brown adipocytes [30]. In obese mice induced by a HFD, the skeletal muscle exhibits reduced oxygen consumption, mitochondrial activity, and AKT protein phosphorylation level. Concurrently, an enlargement of adipocytes in both subcutaneous adipose tissue (SAT) and visceral adipose tissue (VAT) has been noted, and numerous enlarged vacuoles are present in brown adipocytes, indicating metabolic dysfunction and steatosis within the brown adipose tissue [31]. Consequently, the impaired mitochondrial function of skeletal muscle in obesity is accompanied by a decrease in energy and oxygen consumption, which promotes fat accumulation. This, in turn, impairs the thermogenic capacity of brown adipose tissue, further intensifying energy imbalance and obesity.

In summary, obesity, on the one hand, directly triggers an inflammatory response in skeletal muscle via the NF-κB signaling pathway. On the other hand, it activates the AMPK signaling pathway through ectopic lipid deposition, thereby causing mitochondrial dysfunction and, in turn, giving rise to the accumulation of intermediate lipids and aggravating the inflammatory response in skeletal muscle. The combined action of these two aspects affects glucose uptake in skeletal muscle, resulting in skeletal muscle metabolic dysfunction.

## 3. The Development of Skeletal Muscle Fibrosis Caused by Obesity

### 3.1. Pathological Changes

Skeletal muscle tissue fibrosis is a pathological phenomenon characterized by the excessive accumulation of extracellular matrix (ECM) components, especially collagen, which is caused by either excessive production of the ECM, alterations in ECM degradation activities, or both [32]. Myogenic stem cells, namely satellite cells [33], are regulated by fibro/adipogenic progenitor cells (FAPs) [34,35] and play a role in muscle growth [36] and/or repair [37]. In metabolically normal skeletal muscle, paracrine factors derived from FAPs facilitate satellite cell differentiation. Nevertheless, under pathological conditions, the dysregulation of FAPs can result in an increased accumulation of skeletal muscle fiber and adipose tissue [34,38]. Skeletal muscle can conduct energy regulation, adaptation, and remodeling in response to metabolic and nutritional stimuli. However, if pathological fibrosis occurs during the remodeling process, it will limit the plasticity of skeletal muscle tissue and cause it to lose its function.

### 3.2. Impaired Regeneration

Models of obesity resulting from the cytotoxicity of monosodium glutamate to the nuclei of the hypothalamus are widely utilized within the literature [39]. In the obese rat models induced by this approach, reductions in muscle mass, attenuated oxidative capacity, and elevated fibrosis have been identified [40]. Concurrently, a HFD can prompt the diminution of regenerated muscle fibers within skeletal muscle, an increment in collagen fibers, a decrease in the expression of myogenic genes, and an augmentation in the expression of fibrotic genes (including Tcf4, α-Sma, Col1α, and Col3α), thereby indicating that obesity can undermine muscle regeneration [41]. Research has demonstrated that the quantity of PDGFRα-positive FAPs in the skeletal muscle of obese mice is significantly lower, while the number of surviving FAPs in regenerated muscle is relatively higher [41]. This implies that obesity impairs skeletal muscle regeneration by weakening the proliferation of FAPs during the early phase and impeding the clearance of FAPs in the later stage. IL-6, recognized as a paracrine factor of FAPs, can curtail the secretion of insulin-like growth factor 1 (IGF-1) and reduce muscle sensitivity to insulin, thus exerting a negative regulatory effect on the differentiation and growth of muscle fibers [42,43]. Intriguingly, in obese mice fed a HFD [9] and animal models of obesity induced by monosodium glutamate [40], a downward trend in IL-6 expression has been observed in conjunction with the occurrence of skeletal muscle dysfunction.

Collectively, the aforementioned evidence suggests that obesity can impair the muscle regeneration capacity of skeletal muscle and give rise to dysfunctions such as fibrosis, though skeletal muscle might possess a compensatory function during the initial stage.

### 3.3. Mitochondrial Dysfunction

Obesity can result in a decrease in the function of mitochondrial oxidative phosphorylation and an increase in the generation of reactive oxygen species (ROS) [44,45]. Simultaneously, ROS can activate the transforming growth factor-β (TGF-β) signaling pathway, thereby facilitating the activation and proliferation of myofibroblasts [46]. Myofibroblasts are cells with the ability to synthesize and secrete extracellular matrix components, such as collagen. The activation and proliferation of these cells can lead to the excessive deposition of collagen in skeletal muscle, consequently triggering skeletal muscle fibrosis. In the chronic ischemia model of mice [47], it has been observed that obesity can induce mitochondrial pathology and dysfunction in ischemic muscles, accompanied by oxidative stress and inflammation. Concurrently, the level of connective tissue growth factor (CTGF), a potent pro-fibrotic growth factor, increases significantly [47], and it has been demonstrated to promote the excessive accumulation of ECM under hypoxic conditions and in the presence of TGF-β [48,49]. Hence, mitochondrial dysfunction is closely associated with the occurrence of skeletal muscle fibrosis.

### 3.4. Changes in Different Types of Skeletal Muscle

Skeletal muscles in the human body are mainly divided into three types: slow oxidative (Type 1 fibers), fast-twitch oxidative glycolytic (2A fibers), and fast-twitch glycolytic (2B fibers) fibers [50]. The impact of the obese state on different types of muscles in the body is also different, which is reflected in aspects such as metabolic changes and inflammatory responses. IR caused by obesity affects the ability of muscles to take up and utilize glucose. Different types of muscle fibers are affected differently by obesity. Studies have found that after exercise intervention in rats fed with a HFD, insulin-stimulated glucose uptake can be enhanced in all fiber types, except Type 1 fibers [51]. After HFD intervention, almost no change was observed in the expression of glucose transport-related proteins in slow-twitch muscles, while the expression of glucose transport-related proteins in fast-twitch muscles decreased significantly [52]. A HFD can also lead to an increase in the content of intramyocellular lipids (IMCLs) and changes in muscle fiber size. Moreover, in the fast-twitch gastrocnemius muscles, the accumulation of IMCLs is more obvious than that in the slow-twitch soleus muscles [53]. In addition, studies have found that 2B fibers also exhibit an inflammatory phenotype, with NF-κB being one of the core transcription factors [54]. And the inflammatory response can activate immune cells and release cytokines, further exacerbating muscle damage and fibrosis.

During the process of obesity, all three types of fibers will atrophy. However, 2B fibers are larger in volume and have a greater impact on overall muscle atrophy [54]. During the process of obesity, the proportion of oxidative fibers in muscles dominated by oxidative fibers remains unchanged, while the proportion of oxidative fibers in muscles dominated by glycolytic fibers decreases significantly [54].

In conclusion, the occurrence of fibrosis in obese skeletal muscles is closely related to muscle types. Differences in aspects such as metabolism, inflammatory responses, and mechanical stress among different muscle types lead to their varying susceptibilities to fibrosis under obese conditions.

### 3.5. Closely Related to Metabolic Diseases

Diet-induced obesity (DIO) can augment the muscle fat content [9]. Moreover, obesity has been demonstrated to increase the area of adipocytes expressing PERILIPIN and the expression of adipogenic genes (including C-ebpα and PPAR-γ) within regenerated skeletal muscle [41], indicating that obesity induces fat infiltration in skeletal muscle. In the general population of China, an elevation in muscle fat content has been identified to be positively associated with NAFLD [55]. Simultaneously, in a survey of obese individuals, it has been found that patients with non-alcoholic steatohepatitis (NASH) exhibit higher values of skeletal muscle fat infiltration. The degree of muscle fat infiltration is correlated with the high risk of NASH and severe liver fibrosis in severely obese patients [56], suggesting that the fat content in skeletal muscle can serve as a medium-to-high-risk factor for advanced fibrosis in NAFLD.

Sarcopenia, an age-associated disease, is characterized by the progressive loss of muscle mass and function [57]. Skeletal muscle serves as the principal tissue accountable for insulin signaling. In the context of sarcopenia, the reduction of skeletal muscle mass results in diminished insulin signaling, consequently giving rise to IR [58]. IR constitutes a significant mechanism underlying the development of NAFLD and liver fibrosis in sarcopenic obese patients [59,60]. In recent years, the ratio of skeletal muscle mass to the visceral fat area (SV ratio) has been adopted as an index for measuring sarcopenic obesity and has been demonstrated to be closely correlated with cardiometabolic diseases, including T2D, MS, and arterial stiffness [61,62]. Research findings indicate that the negative correlation between the SV ratio and NAFLD is more pronounced in non-obese individuals than in obese participants and that obesity can significantly modify the association between them [63]. Hence, a low SV value represents an indicator that complements traditional obesity measures when evaluating the risk of NAFLD.

**Figure 3:**
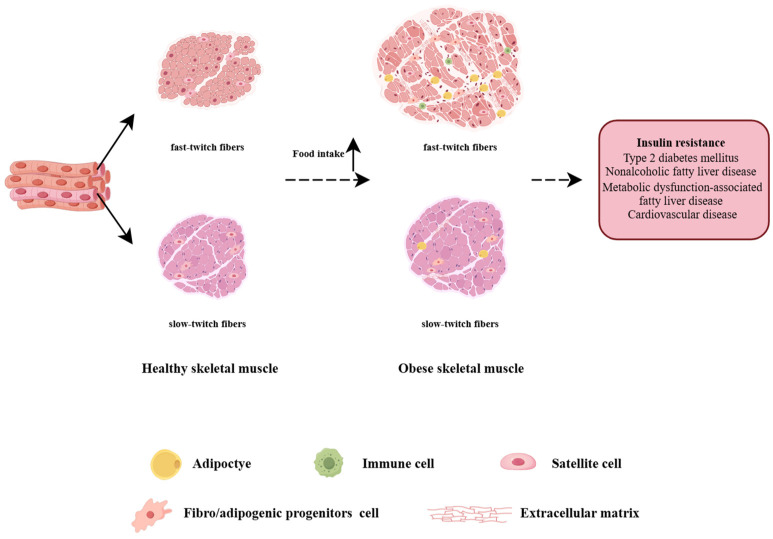
Effects of obesity on different types of skeletal muscle (by Figdraw). Under the condition of obesity, fast-twitch muscles, and slow-twitch muscles will undergo pathological changes to varying degrees, including inflammatory cell infiltration, dysregulation of fibro/adipogenic progenitors, increased intramyocellular lipids, reduced muscle area, and increased production of the extracellular matrix. Skeletal muscle fibrosis causes its dysfunction and is closely associated with the occurrence of various metabolic diseases, such as insulin resistance, type 2 diabetes, non-alcoholic fatty liver disease, metabolic dysfunction-associated fatty liver disease, and cardiovascular diseases.

Therefore, obesity can impair the regenerative ability and mitochondrial function of skeletal muscle, leading to an increase in collagen fibers and intramuscular fibrosis, which will disrupt the glucose uptake function, trigger IR and metabolic disorders, and different types of skeletal muscle also undergo distinct changes. Meanwhile, obesity can result in an increase in lipid infiltration in skeletal muscle, a decline in muscle mass and function, and an increase in the morbidity and mortality of complications such as NAFLD. In summary, obesity can cause the occurrence of skeletal muscle fibrosis and is closely related to the body’s metabolic diseases (as shown in Figure 3).

## 4. The Association Between Inflammation and Fibrosis in Skeletal Muscle Caused by Obesity

### 4.1. Changes in Inflammatory Factors: The Close Association with the Occurrence of Fibrosis

Many studies have found that skeletal muscle inflammation and fibrosis caused by obesity coexist, and they usually occur simultaneously. In the DIO model of rats fed with a high-fat/high-sucrose (HFS) diet, concurrent augmentations in intramuscular lipids, inflammatory cells, and muscle tissue fibrosis within the quadriceps femoris have been identified [64]. Furthermore, obese mice on a HFD exhibit an upward trend in the expression of p-NFκB and TGFβ1 [9]. Experimental evidence has shown that in the gastrocnemius muscle of obese mice induced by a HFD [13], the mRNA expression levels of inflammatory factors including TNF-α, TLR2, TLR4, and MCP1 are elevated. This elevation is concomitant with a reduction in the size and number of muscle fibers and an up-regulation of the expression of atrophy factors such as FoxO1, FoxO3, MuRF1, and atrogin1. The elevation of the TNF-α level has been demonstrated to be associated with decreased insulin sensitivity, enhanced muscle catabolism, sarcomere ubiquitination, and NADPH oxidation [42,43]. Collectively, these alterations may exert a negative influence on muscle development and differentiation. In the obese animal model induced by glutamate, an increase in plasma TNF-α secretion has been observed, along with a diminished availability of the paracrine effect of MyoD. This results in a decreased differentiation of myoblasts into myocytes, a reduction in myotube fusion [40], and a decline in skeletal muscle function. Moreover, various inflammatory cytokines, namely C-reactive protein (CRP), IL-6, interleukin-10 (IL-10), interleukin-15 (IL-15), and TNF-α, have been verified to be responsible for the reduction in muscle mass and the increase in macrophage infiltration, thereby facilitating the transformation of skeletal muscle into a fibrotic state [65,66]. Accordingly, changes in inflammatory factors in obese skeletal muscle play an important role in the process of fibrosis development. However, there is still a lack of evidence regarding both the sequence of occurrence of the two and the existence of mutual regulation between them; thus, a large number of studies are still needed to explore the specific molecular mechanisms involved.

### 4.2. Co-Regulation of Adipose–Liver–Skeletal Muscle Tissue Network

Some studies have pointed out that there is a complex inter-tissue and intra-tissue co-expression network among skeletal muscle, adipose tissue, and the liver. Obesity results in chronic, low-grade, systemic inflammation, and the adipose tissue and skeletal muscle of obese patients both present a chronic low-grade inflammatory state. Experiments have demonstrated that T cells and macrophages residing in extramyocellular adipose tissue (EMAT) of skeletal muscle cells can induce the expression of pro-inflammatory chemokines, thereby expanding the inflammatory response and IR of skeletal muscle through the Janus kinase/signal transducer and the activator of the transcription (JAK/STAT) signaling pathway [67]. The adipokine WISP1 has also been proven to promote skeletal muscle IR through the TLR4-activated inflammation/c-Jun N-terminal kinase (JNK) signaling pathway [12]. It illustrates that under the condition of obesity, the changes among various tissues are not isolated, but rather influence and regulate each other.

It has been demonstrated that the occurrence of liver fibrosis is associated with the activation of the NF-κB inflammatory signaling pathway. Through the elevation of the expression of inflammatory factors, this activation augments the level of oxidative stress within the liver, exacerbates hepatocyte damage, and triggers the activation of hepatic stellate cells (HSC) [68]. Consequently, a substantial deposition of collagen-based ECM in the liver tissue ensues, leading to the formation of fibrosis [69], which indicates that the inflammatory response in the liver regulates the occurrence of fibrosis. Moreover, inflammation in adipose tissue and skeletal muscle induced by obesity has also been verified to be closely linked to liver fibrosis [66]. A cohort study revealed that the concurrent presence of metabolic-associated fatty liver disease (MAFLD) and sarcopenia is associated with significant fibrosis and is also strongly correlated with a remarkable increase in the risk of death related to cardiovascular diseases and diabetes [70]. This finding implies that sarcopenia is associated with a high mortality risk in MAFLD. These pieces of evidence once again confirm the situation that the adipose–liver–skeletal muscle tissue network coordinately regulates metabolic activities.

Given that these three tissues simultaneously serve as target organs for glucose uptake and pathophysiological processes such as inflammation, ectopic lipid deposition, oxidative stress, and fibrosis occur in all of them under the condition of obesity, there is a co-expression network regulating metabolic activities. Thus, we speculate that there is an inflammation-regulated fibrosis process within the co-expression network. Obesity leads to the overexpression of inflammatory factors like TNF-α, resulting in a systemic inflammatory state. In skeletal muscle, it exacerbates the inflammatory response by activating the NF-κB signaling pathway and causes muscle regeneration impairment, collagen fiber deposition, fibrosis, and other dysfunctions through ectopic lipid deposition and mitochondrial oxidative stress. Subsequently, it triggers metabolic disorders such as IR, T2D, and NAFLD. Meanwhile, the pathological changes in adipose tissue and the liver also have an impact on the metabolic function of skeletal muscle. However, the evidence regarding skeletal muscle inflammation and fibrosis under the condition of obesity is still insufficient, and, to a large extent, the regulatory relationship between them remains unclear. Our speculation put forward based on the existing evidence requires more research to confirm its reliability.

## 5. Intervention Strategies

### 5.1. Aerobic Exercise

Aerobic exercise exerts a positive influence on the inflammatory response within skeletal muscle and the insulin-signaling pathway. Research indicates that aerobic exercise can significantly decrease the expression levels of inflammatory factors, such as TNF-α and IL-6, in the skeletal muscle of obese rats [71]. Simultaneously, aerobic exercise can enhance the insulin-signaling pathway by increasing the expression of phosphorylated AKT and GLUT4, while reducing the expression of protein tyrosine phosphatase 1B. This, in turn, augments the uptake and utilization of glucose in skeletal muscle and alleviates IR [29]. Furthermore, exercise training has been demonstrated to increase the cross-sectional area of muscle fibers and the number of satellite cells dedicated to the myogenic lineage as well as improve the morphology of irradiated muscles and satellite cell dynamics [9].

Therefore, aerobic exercise can improve both inflammation and fibrosis of skeletal muscle in obesity, protect the function of skeletal muscle, and is of great significance for the maintenance of systemic homeostasis.

### 5.2. Dietary Modification

Fructose plays a significant role in skeletal muscle inflammation and fibrosis induced by obesity. Researchers have demonstrated that fructose can influence skeletal muscle health via multiple pathways. Over 50% of fructose is metabolized via fructolysis in the liver [72]. Excessive fructose intake can up-regulate the carbohydrate response element-binding protein (ChREBP) in the liver, which activates glucose-6-phosphatase (G6Pase) and enhances gluconeogenesis to convert it into glucose [73]. Meanwhile, part of it is converted into lactic acid and released into circulation, and the production of intermediate products in the tricarboxylic acid cycle is increased to promote the synthesis of fatty acids. Glucose generated from glucose-6-phosphate hydrolyzed by G6Pase in the liver [74] will be transported out of liver cells through glucose transporter type 2 (GLUT2) and then enter the bloodstream [75,76], and be transported to tissues and organs throughout the body. Fructose can induce hyperlactatemia, thereby inhibiting GLUT4, reducing the uptake and utilization of glucose in skeletal muscle, and leading to IR [72]. A high-fructose diet can induce myocardial lipid accumulation, fibrosis, and inflammatory responses in mice, suggesting a potential connection between fructose and inflammation and fibrosis [77]. Meanwhile, fructose can promote the accumulation of skeletal muscle fat by increasing lipid synthesis and lipid transport in skeletal muscle, resulting in skeletal muscle inflammation and fibrosis. For example, long-term intake of fructose and glucose significantly increased the lipid content in the skeletal muscle of rats, and the level of the pro-inflammatory factor IL-6 in the blood and skeletal muscle also increased significantly [64,78].

Therefore, controlling fructose intake is crucial for preventing skeletal muscle inflammation and fibrosis induced by obesity. Reducing the consumption of high-fructose foods, such as sugary drinks and processed foods, can decrease the adverse effects of fructose on skeletal muscle.

### 5.3. Pharmacotherapy

Currently, most of the targeted therapy research on skeletal muscle metabolic dysfunction mainly focuses on aspects such as anti-inflammatory, anti-oxidative stress, and anti-fibrosis-inducing factors. Empagliflozin has been demonstrated to enhance the phosphorylation of AMP-activated protein kinase and acetyl-CoA carboxylase within skeletal muscle, as well as increase the level of fibroblast growth factor 21 in plasma [79]. By facilitating fatty acid oxidation in skeletal muscle, it can reduce tissue fibrosis and IR resulting from ectopic fat deposition. Moreover, attenuating the phosphorylation of JNK and ERK1/2 can alleviate obesity-induced inflammation and metabolic alterations.

Furthermore, in animal experiments, it has been demonstrated that luteolin (LU) can enhance muscle mass and function. This is achieved by suppressing the degradation of proteins within adipose tissue, plasma, and muscle in vivo, as well as by mitigating lipid accumulation and inflammation [13]. CDDO-ethyl amide (CDDO-EA) has also been verified to impede the expression of endotoxin-induced TNF-α and MCP-1 genes through the inhibition of the NF-κB signaling pathway in rat myotube cells [80]. Consequently, this suppresses the inflammatory responses of macrophages and myocytes. Moreover, in the soleus muscles of mice supplemented with fucoxanthin (FCX), a significant augmentation in the expression of genes associated with mitochondrial biogenesis and fatty acid β-oxidation has been noted [81]. This is accompanied by an improvement in skeletal muscle function, enhanced mitochondrial function, reduced fat accumulation, decreased reactive oxygen species generation, and alleviation of chronic inflammation [82,83]. Retinoic acid (RA) can augment the proliferation of FAPs during the early phase of skeletal muscle regeneration. Simultaneously, during the remodeling stage, RA can inhibit the differentiation of FAPs and promote apoptosis [41]. Supplementation of RA in obese mice not only rescues the impaired muscle fiber regeneration but also restrains the infiltration of fat and fibrotic tissues during the muscle repair process. Collectively, these studies furnish a robust theoretical foundation for exploring the mechanism underlying the occurrence and development of obesity-related skeletal muscle inflammation and fibrosis. Additionally, they present potential targets for the treatment of metabolic diseases.

Lifestyle modifications, dietary adjustments, and drug interventions can exert their effects on aspects such as inflammatory responses, lipid deposition, oxidative stress, and factors that induce fibrosis. Through these means, skeletal muscle function can be enhanced and IR can be improved. Nevertheless, currently, the majority of the treatments for obese skeletal muscle dysfunction are mainly confined to short-term animal experiments or small-scale clinical studies, lacking evidence from long-term clinical follow-up. Further research on specific molecular mechanisms and long-term clinical studies are still required. Consequently, in the future, the “obesity-targeted” anti-inflammatory and anti-fibrosis treatments for metabolic diseases, along with the exploration of personalized intervention strategies, will continue to be the direction that we strive for.

## 6. Discussion

This study has conducted an in-depth exploration of the relationship between skeletal muscle inflammation and fibrosis resulting from obesity. Firstly, the process of obesity development is accompanied by the occurrence of inflammatory responses and tissue fibrosis remodeling. Skeletal muscle fibrosis is triggered by the energy balance disorder caused by obesity, and, simultaneously, it exacerbates obesity-related metabolic disorders. Obesity can induce a chronic inflammatory state in skeletal muscle, which leads to mitochondrial oxidative stress, an increase in fatty acids, and an elevation in the secretion of inflammatory factors. Subsequently, these changes will activate the inflammatory signaling pathways. Along with the progression of fibrosis, they will lead to skeletal muscle dysfunction, impair glucose uptake ability, reduce metabolic function, and further contribute to the emergence of metabolic diseases such as IR, diabetes, and NAFLD. Hence, the prevention of skeletal muscle dysfunction constitutes an effective strategy for improving energy metabolism and blood glucose balance.

To date, the majority of our current knowledge has been derived from animal studies. Although animal models are of critical importance for understanding the biological properties of skeletal muscle, it remains to be determined whether the metabolic regulation within these models parallels that in humans. Given that our ultimate objective is to apply these findings to human diseases, the research conclusions obtained thus far require further verification in human subjects. Additionally, most existing research has primarily focused on demonstrating the close association between obesity and skeletal muscle inflammation. Specifically, inflammation is accompanied by muscle atrophy, fibrosis, and poor function at the muscle cell level. However, the relevant regulatory mechanisms have not been clearly described, and it remains unknown whether these processes regulate the occurrence of sarcopenia, fibrosis, diabetic muscle dysfunction, and chronic muscular myopathy atrophy.

Currently, intervention strategies for obesity mainly encompass exercise intervention, dietary regulation, and bariatric surgery, while effective drugs targeting associated complications are yet to be developed. Aerobic exercises can ameliorate skeletal muscle inflammation and fibrosis, and, furthermore, controlling fructose intake and maintaining a balanced diet are also vital for preventing obesity-induced skeletal muscle inflammation and fibrosis. It has been thus identified that both pharmacological and lifestyle interventions can improve skeletal muscle function, potentially through anti-inflammatory effects, reducing the incidence of fibrosis and improving IR. In summary, skeletal muscle inflammation and fibrosis caused by obesity represent complex processes involving multiple mechanisms. Through an in-depth investigation of these mechanisms, we can gain a better understanding of the pathogenesis of obesity-related diseases. This, in turn, inspires us to focus on improving skeletal muscle inflammation and maintaining skeletal muscle plasticity, thereby providing novel perspectives for the treatment of skeletal muscle fibrosis caused by obesity. Based on the relationship between inflammation, fibrosis, and obesity-related metabolic dysfunction, anti-inflammatory and anti-fibrotic approaches are also regarded as promising treatment strategies for obesity-related metabolic disorders.

Future research could further explore the following aspects. Firstly, an in-depth examination of the specific mechanisms of action of different cells and molecules in skeletal muscle inflammation and fibrosis induced by obesity aims to provide targets for the development of targeted therapeutic drugs. Secondly, the exploration of more personalized intervention strategies to formulate the most suitable exercise and diet regimens based on factors such as the degree of obesity, physical condition, and genetic background of individuals. Third is conducting long-term clinical studies to verify the preventive and therapeutic effects of exercise and diet interventions on skeletal muscle inflammation and fibrosis in obese patients, thereby providing more reliable evidence for clinical practice.

## 7. Conclusions

This review clearly shows that there are intricate and close connections between obesity, skeletal muscle inflammation, and fibrosis. Among them, inflammation most likely plays a key role in regulating the occurrence and development of fibrosis. However, at present, the specific occurrence and development mechanisms of the two have not been fully clarified and still need to be further explored in depth. Analyzing the pathogenesis of obesity more deeply is expected to explore new targets for the treatment of metabolic diseases, thus bringing new breakthroughs and hopes to the relevant medical fields.
